# Experimental and Theoretical Study on the Homodimerization Mechanism of 3-Acetylcoumarin

**DOI:** 10.3390/molecules27217228

**Published:** 2022-10-25

**Authors:** Kristina B. Simeonova, Ana I. Koleva, Anna-Mariya R. Zlatanova, Nevena I. Petkova-Yankova, Hristiyan A. Aleksandrov, Petko St. Petkov, Rositca D. Nikolova

**Affiliations:** Faculty of Chemistry and Pharmacy, Sofia University “St. Kliment Ohridski”, 1 James Bourchier Blvd, 1164 Sofia, Bulgaria

**Keywords:** reaction mechanism, homodimerization, 3-acetylcoumarin, density functional theory calculations, coumarin, DFT modeling, 2-oxo-2*H*-1-benzopyran

## Abstract

In the present study, the reaction conditions for homodimerization process of 3-acetylcoumarin were achieved under sonication using combination of zinc and metallic salt (ZnCl_2_ or Zn(OAc)_2_). Appropriate frequency and sound amplitude have been identified as significant variables for the initiation of the reaction. On the base of first principal calculations and experimental results, the mechanism of the reaction was investigated. The relative stability of the possible intermediates has been compared, including evaluation on the ionic and radical reaction pathways for the dimerization process. Theoretical results suggested that the radical mechanism is more favorable. The C-C bond formation between the calculated radical intermediates occurs spontaneously (∆G = −214 kJ/mol for ZnCl_2_, −163 kJ/mol in the case of Zn(OAc)_2_), which proves the possibility for the homodimerization of 3-acetylcoumarin via formation of radical species. Both experimental and theoretical data clarified the activation role of the solvent on the reactivity of the Zn-salt. The formation of complexes of solvent molecules with Zn-atom from the ZnCl_2_ reduces the energy barrier for the dissociation of Zn-Cl bond and facilitate the formation of the dimeric product.

## 1. Introduction

Coumarins are compounds with pharmacological functions and application as drugs for broad range of diseases [1,2,3]. They could be involved in different reaction conditions defining the formation of ionic, radical or radical anionic intermediates [4,5,6,7]. The behavior of the aromatic system frequently supports an electron transfer processes and characterized the substituted coumarins as antioxidants. Usually, these reactions are promoted by electroreduction, photochemical initiation or utilization of Grignard reagents [8,9,10,11,12,13,14,15,16].

Modeling biscoumarin systems containing two moieties of the same or different types has recently been our focus [17]. Moreover, such compounds exhibit properties similar to the coumarins which they were built from; for example, anti-coagulants, anti-oxidants, anti-tumor and anti-fungal [18,19]. Their biological activity as inhibitors of enzymes (anti-HIV-1 protease and integrase, DNA polymerase, protein kinase and urease) [20,21,22,23,24] is not well studied.

**Scheme 1:**
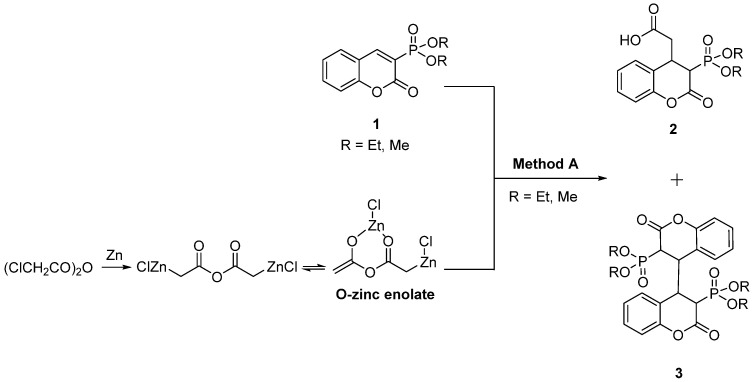
Reaction of coumarins **1** with chloroacetic anhydride under ultrasound irradiation.

Previously, we have reported the formation and characterization of the biscoumarins **3** (Scheme 1) as a result of a dimerization of 2-oxo-2*H*-1-benzopyran-3-phosphonic acid **1** in the presence of metallic zinc and chloroacetic anhydride under ultrasound irradiation [17]. The procedure is rapid and simple to perform characterized by excellent yield for the isolated dimers. Previous assumption for the reaction mechanism was an initiation of the process by formation of a dizinc reagent. Compounds **3** were products of a dimerization process in which two identical coumarin fragments were directly linked at their C-4 atoms.

**Scheme 2:**
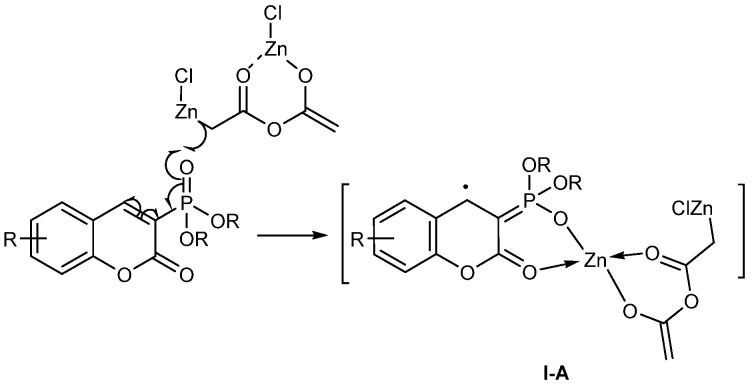
Possible radical formation from compounds **1**.

Based on the obtained results [17], we assumed that the most probable reaction path for the formation of biscoumarins includes a radical initiation assisted by the substituents in third position. It was reasonable to presume that the activation of the C3=C4 bond in the studied systems was responsible for the formation of the dimeric coumarin structures. We believed that the activation could also depend on the substituent’s electronic effects at the benzene nucleus, which could favor or destabilize the intermediate **I-A**, Scheme 2, reported previously.

**Scheme 3:**
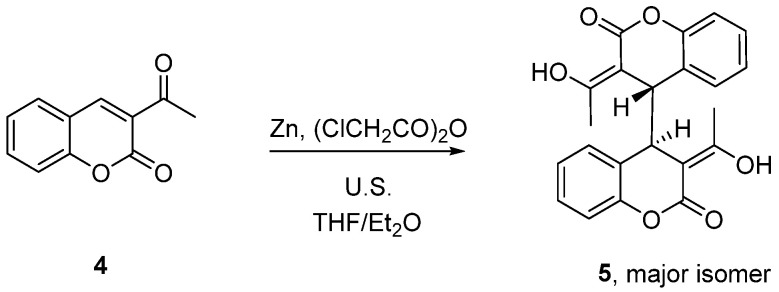
Dimerization of coumarin **4** under ultrasound irradiation.

Herein we report our experimental and theoretical observations on the dimerization of 3-acetylcoumarin **4**, Scheme 3. In this example, the 3-substituted coumarin is bearing enolizable acetyl group and affords fast reaction initiation. Many factors could play a role on the outcome of the reaction—different initiators, metals, reagent ratio, reaction media, ultrasound frequency, sound amplitude, etc. In order to highlight the most plausible reaction pathway, density functional theory (DFT) calculations were used for comparison of different type of mechanisms and relative stability of radical or ionic species that could be formed in the studied reaction conditions [25,26,27]. Combining first principal calculations and the extended experimental data on the homodimerization reaction provided us arguments for the validation of the mechanism.

## 2. Results and Discussions

### 2.1. Experimental Conditions—Elucidation and Modification of the Homodimerization Reaction

As part of our continuing efforts to obtain differently substituted coumarins, we firstly tried to study and modify the previously applied conditions for the dimerization of the 3-acetylcoumarin **4**, thus trying to optimize an atom-economy conditions for the dimerization process, Scheme 3. Therefore, firstly the outcome of the reaction was tested only by altering the amount of the used chloroacetic anhydride, Table 1.

The reaction was carried out in a THF/Et_2_O media under ultrasound irradiation conditions. Different ratios between the coumarin **4** and chloroacetic anhydride ((ClCH_2_CO)_2_O) were used while the amount of the metallic zinc was kept constant—5.6 eqv. The complete conversion of the monomer **4** to product **5** took place in a short time (10–15 min) with high yield (90%) in a ratio of coumarin to (ClCH_2_CO)_2_O 1:1.5. Thus, using nearly half of the previously needed amount of the anhydride, the result from the reaction is almost the same. Therefore, respecting the molecule economy principles, the 1:1.5 ratio of the reagents was used in the further experiments.

To clarify the role of the used ultrasonic irradiation on the dimerization process, different ultrasound frequencies (20 kHz, 37 kHz, and 80 kHz) and sound amplitudes were used at constant temperature (40 °C). The results from the study are presented in Table 2. As it could be seen, there is a significant importance of the used ultrasonic irradiation for the sonochemical change. The dimerization reaction was performed for shorter time and better yields when high powered 37 kHz frequency was used.

It should be noted that the yields for the dimer increase with the decreasing of the wave amplitude. Even when the low powered frequency was applied (80 kHz) an activation of the coumarin system is observed. However, a longer reaction time was needed for the complete conversion of 3-acetylcoumarin. In all the performed experiments, the product’s yield remained high, yet not so in the case of 20 kHz ultrasonic bath (10 min, 92%) [17]. The results showed an activation maximum around 60% of the amplitude applying low power whether high power frequency cover the conditions for the maximum at 30%. Additionally, the obtained results for 37 kHz and 30% wave amplitude are comparable to this with 20 kHz ultrasonic bath.

Furthermore, we could assume that the frequency and the amplitude of the ultrasonic bath play a major role and most probably it influences the activation of the zinc metal. In order to better understand the homodimerization reaction, we set out to explore a cost-effective synthetic protocol for the formation of biscoumarins by applying conditions where the initiator of the reaction is a combination of metal and metal salt. Moreover, the role of the reagents in the initiation step as well as the nature of the intermediate of the dimerization are valuable for the mechanism determination.

**Scheme 4:**
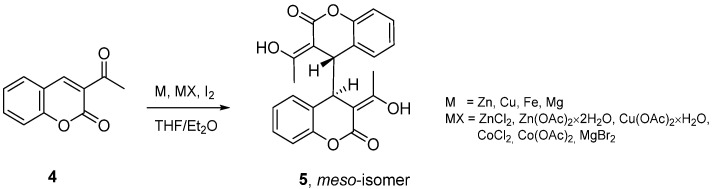
Initiation of the reaction by different metals and metal salts.

To address the above-mentioned study, we firstly sought to find a suitable transition metal for the reaction, Scheme 4, Table 3. The metals that were tested as initiators—Zn, Cu, Fe, and Mg were chosen due to their application as catalysts in electron transfer processes [11,12] and in some cross-coupling reactions [28,29,30]. Moreover, during electron transfer conditions, radical intermediates which could form the dimeric products were reported. Zinc, copper, magnesium, and cobalt salts were chosen to be tested in the reaction due to the good chelating properties of the metal core and the possibility for stabilization of the intermediate by forming a chelate complex was considered as well. All the listed experiments were performed in a 37 kHz ultrasonic bath with 30% amplitude and the reaction mixture was heated to 40 °C.

The homodimerization reaction of 3-acetylcoumarin **4** was initially performed by applying zinc chloride. The zinc salt was chosen due to the possibility of its formation as a side product in the reaction with (ClCH_2_CO)_2_O and the possibility to be the actual catalyst of the process. Additionally, the ZnCl_2_ is a Lewis acid that by the assistance of the acetyl group in position C-3 could activate the studied coumarin **4**. The zinc acetate (Zn(OAc)_2_) was used as an analogue of ZnCl_2_ with better solubility in organic solvents.

As could be seen from Table 3, electron transfer conditions combining Zn and zinc salt were appropriate to activate the coumarin species for the dimerization reaction. The dimeric structure was obtained in quantitative yields (97%, **Method B.4**) while maintaining the amount of the used metal of 5.6 eqv., relative to the starting 3-acetylcoumarin, and increasing the amount of the used zinc acetate from 1.5 to 3.0 eqv. The reaction was carried out in THF/Et_2_O media with similar conditions to the ones used with chloroacetic anhydride. Comparing to the conditions listed as **Method A** including chloroacetic anhydride the reaction time was kept relatively short. The reactions assisted by ZnCl_2_ (**Method B.1** and **Method B.2**) were also successful; however, the yields were 30% lower compared to the zinc acetate under the same conditions. It is interesting to note that even in the presence of an oxidant (**Method B.9**), the homodimerization product could be isolated in high yield. However, the reaction time was substantially longer.

Other metal salts (copper bromide, copper acetate, cobalt chloride, and cobalt acetate) were tested as Lewis acid analogues of ZnCl_2_ and Zn(OAc)_2_, **Method B.5–B.8**. The dimeric product was isolated only when the reaction was performed with cobalt chloride, **Method B.8**, even though the full conversion of the starting coumarin was detected after one day.

The ability to obtain the biscoumarin product **5** was studied in the presence of different metals—copper, magnesium and iron, **Method B.11-B.14**. As catalysts zinc (II) or copper (II) acetate and anhydrous magnesium chloride were applied in the reaction. However, the dimerization process was not initiated, Table 3. It is interesting to note that when metallic zinc was added to the reaction mixture of **Method B.11** a complete consumption of the starting 3-acetylcoumarin was detected after 240 min. Therefore, we could assume that the presence of metallic zinc is essential for the homodimerization reaction of 3-acetylcoumarin **4** in both conditions—including the anhydride [17] and the metal salts.

As could be seen, the reaction is sensitive to several effects and its outcome cannot be estimated so easily. The different solubility of zinc (II) salts, compared to chloroacetic anhydride, demanded an optimization of the organic solvents or mixture of solvents that could be used in the reaction (Table 4).

Benzene and dichloromethane were the first two solvents that were tested due to their weak solvating ability towards radicals and ionic species; therefore, they could accelerate the dimerization process. Unfortunately, benzene as a reaction media was not suitable for the process. Despite the long reaction time (80 min) which was probably due to the poor solubility of the used catalyst (zinc (II) acetate), methylene chloride was appropriate for the dimerization, however, a complexed mixture was observed and it was hard to isolate the desired product in pure form. Thus, it prompts us forward to try different polar solvents that could solvate the intermediate as well as stabilize it. The most common solvents for the organometallic reactions—diethyl ether and tetrahydrofuran (Et_2_O, THF), were our next choice. In the studied reaction, THF was the solvent that accelerated the reaction rate with conversion time for compound **4** of only 10 min. Surprisingly, the reaction did not occur in Et_2_O. A possible assumption for the observed results is the different solvation of the species formed during the reaction. It can be implied that in this study THF is most suitable solvent for single-electron-transfer reactions.

The next investigations were directed to the optimization of the amount of metallic Zn and the zinc salt relative to the quantity of the starting 3-acetylcoumarin **4.** The findings were important in terms of the atom-economy ratio determination of metal and the starting coumarin. The influence of the zinc salt is essential for the reaction initiation and for the formation of the chelated intermediate complex as well. Initially, the reactions were carried out by varying the amount of the metal while the amount of the catalyst was kept constant (3 eqv.), Table 5.

The results in Table 5 indicate that the optimal amount of zinc needed to carry out the dimerization reaction is 4 eqv. relative to the starting 3-acetylcoumarin **4**, **Method C.4**. Full conversion of the coumarin **4** was observed only for 10 min, and the product **5** was isolated with 77% yield. The lower results obtained with 1 and 2 eqv. of metal support our previous hypothesis that a larger metal surface is needed for successfully performing the dimerization reaction, **Method C.1** and **Method C.2**. Moreover, the dimerization process was slowed down (90 min for full conversion), and the yields were 65% and 64% respectively. The usage of larger amounts of zinc, **Methods C.5–C.7**, also did not lead to higher yields (62–69%). TLC monitoring of the reaction mixture in these cases showed formation of side reaction products.

Another factor that could play a major role on the outcome of the reaction is the amount of the applied salt when the combination metal/metal salts is used. The optimization of the used catalyst’s amount, Zn(OAc)_2_ × 2H_2_O, was performed in relation with the results listed in Table 3. For example, when the amount of zinc salt was increased to 3 eqv., the rate of dimerization also increased as well as the yield of the biscoumarin product. Initially, the reactions were carried out by varying the amount of the metal salt while the amount of the metallic zinc (4 eqv.) and the reaction media (THF) was kept constant, Table 6.

It should be noted that no reaction was detected when the ratio between the coumarin and the zinc salt was 1:1, **Method D.1**. While the amount of the used salt was increased to 2 eqv., **Method D.2**, the reaction took place for a longer time compared to the conditions listed as **Method D.3**. However, additional increase of the amount of the used salt did not improve the yield of the product. Thus, it made the isolation of the dimeric structure more difficult due to the formation of side products. From this set of experiments, we could assume that the optimal quantity of zinc acetate is 3 eqv. and the nature, the amount of the used metal and metal salts play a major role for the outcome of the homodimerization reaction.

All the experimental findings, discussed above, emphasize the importance of the metal and the metal salt for the successful coumarin dimerization reaction. Besides that, the mechanism of the reaction and the synergism between the metal and the metal salt is not well clarified. Similar structures were obtained via electrochemical reduction and photochemical conditions where radicals/radical-anions formation were observed [11,31]. By means of density functional theory, we studied several reaction pathways, including radical and ionic species formation, and compared their relative stability (see Figure S2).

We have initially investigated the possibility of formation of two different chelated complexes. In the first case M-C bond is formed between metal and C atom in position 4, where C-C bond would be formed later. In the second type of complexes metal center is coordinated to two O-atoms: one from the lactone ring and one from the acetyl group.

### 2.2. Activation Effect of the Solvent

**Figure 1:**
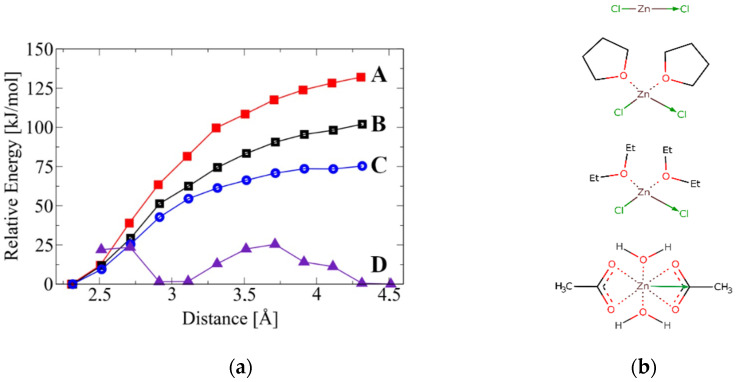
Bond dissociation energy: (**a**) Changes in the relative energy of the metal salts, with respect to the Zn-Cl distance of ZnCl_2_ and Zn-carbonyl C-atom of Zn(OAc)_2_ × 2H_2_O: **A**—Zn-Cl bond dissociation energy in PCM(THF); **B**—Zn-Cl bond dissociation energy of ZnCl_2_x2Et_2_O in PCM(Et_2_O); **C**—Zn-Cl bond dissociation energy of ZnCl_2_ × 2THF in PCM(THF); **D**—Dissociation of acetyl anion from Zn(OAc)_2_ × 2H_2_O in PCM(THF); (**b**) Schematic representation of the structures of the investigated molecules for the bond dissociation energy. The arrows denote the corresponding reaction coordinate.

In order to check the stability of the metal salts with coordinated solvent molecules, the dissociation energy of Zn-Cl bond in the molecule of ZnCl_2_, with and without coordinated solvent molecules was investigated, Figure 1. The bond dissociation energy was estimated on the base of the relaxed potential energy scan of the Zn-Cl bond. The results indicate that explicit coordination of two Et_2_O or two THF molecules to the Zn in the ZnCl_2_ salt reduces the Zn-Cl bond dissociation energy up to 52 kJ/mol with respect to bare ZnCl_2_, thus pre-activate the metal salt reactivity.

The observed theoretical data is in compliance with the experimental findings where no reactions were detected in Et_2_O. We have also investigated the dissociation energy of the AcO-group in the molecule of Zn(OAc)_2_ × 2H_2_O by increasing the distance between the Zn-atom and the C-atom from the acetyl group. From these calculations, it can be seen that the formation of an acetate anion proceeds via two-step process, thus requires less energy (see Figure S3) than Zn-Cl bond dissociation. These findings are in line with the experimental observations, the reaction takes place for shorter time and higher yield in Zn(OAc)_2_ × 2H_2_O than in ZnCl_2_.

**Scheme 5:**
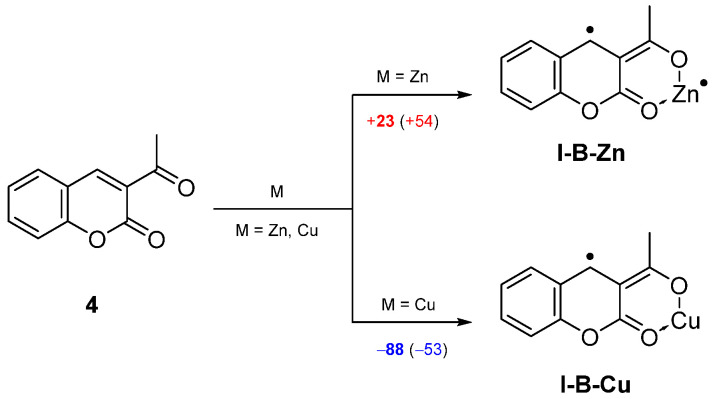
Schematic representation of the initiation of the reaction with different metals. The energies for the formation of the intermediates’ **Enthalpies** (Free energies given in parenthesis) are presented in kJ/mol.

**Scheme 6:**
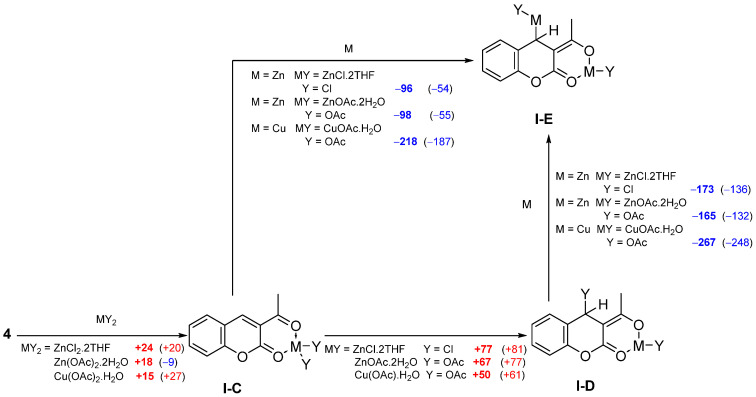
Schematic representation of the ionic reaction mechanism. The **Enthalpies** (Free energies given in parenthesis) for the formation of the intermediates are presented in kJ/mol.

The relative stability of various possible complexes between the monomer and the ZnCl_2_ were compared and, as reported in the literature [32], the structure that has the ZnCl_2_ chelated to both O-atoms (from the lactone ring and the acetyl group) is more stable, due to the 6-membered cycle that is being formed—intermediate **I-C** (see Table S1). The theoretical study of the mechanism of 3-acetylcoumarin dimerization could be divided by nature of the intermediates—ionic and radical. First, we will discuss the radical formation (Scheme 5), followed by stability of the ionic intermediates (Scheme 6).

As it is known from experimental data, the presence of not only metal salt but also the metal itself, during the reaction, is essential. Therefore, we have also considered the possibility of a coordination of the metal atom to the molecule of the 3-acetylcoumarin **4** in different positions (coordination towards the double bond between C3 and C4 atoms in the coumarin and coordination towards the oxygen atoms).

After finding the most energetically favorable intermediates that could be formed during the reaction process, we have constructed a plausible reaction scheme that represents several reaction pathways, leading to the formation of the desired product—the homodimer of the 3-acetylcoumarin **5**. The reaction scheme is focused on both possibilities—the mechanism being either ionic or radical, Figure S2. In the reaction scheme we have considered the possibility of the intermediates forming with different metals (Zn and Cu) and different metals salts (ZnCl_2_, Zn(OAc)_2_, Cu(OAc)_2_). We know from experimental data that the reaction conditions, including those with Zn as a metal, give good yields and if only Cu metal and salts are used, a product is not observed. This is the reason we choose these two border cases in the quantum chemistry modeling. All the calculations were conducted in implicit solvent (PCM) THF and we have also considered the possibility of the formation of complexes between the metal salts and solvent molecules. The chelated complexes of the 3-acetylcoumarin and the metal salt show higher thermodynamic stability with respect to the non-chelated ones. Thus, we have used those complexes for further investigation of the reaction pathway. Several different reaction pathways for the formation of the intermediates, that could potentially lead to the homodimerization of 3-acetylcoumarin **4**, have been considered (see Figure S2).

### 2.3. Ionic Mechanism

**Scheme 7:**
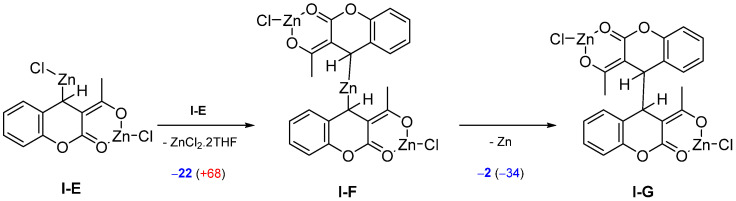
Schematic representation of the ionic reaction mechanism. The **Enthalpies** (Free energies given in parenthesis) for the formation of the intermediates are presented in kJ/mol.

In the case of the ionic mechanism, we have considered the formation of intermediates **I-C**, **I-E**, **I-D,** and **I-F** (Scheme 6 and Scheme 7). From the proposed several reaction pathways, the organometallic intermediate **I-E** takes part in each of the reaction steps towards formation of the product. For this reason, we hypothesize that its formation and presence in the reaction mechanism is essential for the formation of the homodimer following the ionic reaction mechanism.

One of the possibilities for the formation of the homodimer is the formation of C-C bond from intermediates **I-C** and **I-E**. The calculated energy for this reaction is about −20 kJ/mol (ZnCl_2_) and −34 kJ/mol (Zn(OAc)_2_), respectively, which makes it a plausible reaction pathway. Moreover, we found that formation of **I-E** from **I-C** is an exergonic process. Hence, the formation of intermediate **I-E** from intermediate **I-C** is a spontaneous process, the possibility of two molecules from intermediate **I-E** forming the desired product is not excluded. In this scenario, the formation of the final product **I-G**, proceeds via formation of intermediate complex **I-F**. The formation of the intermediate **I-F** from **I-E** is slightly exothermic (∆H = −22 kJ/mol), while if we take into account the entropy contribution, the reaction becomes endergonic (∆G = +68 kJ/mol), Scheme 7.

**Scheme 8:**
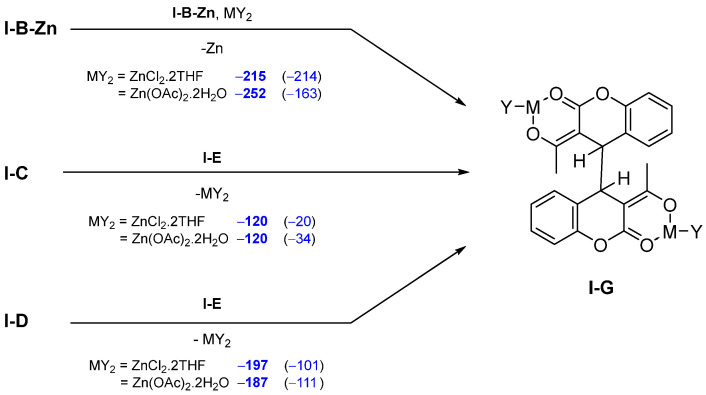
Schematic representation of the reaction mechanism. The **Enthalpies** (Free energies given in parenthesis) for the formation of the dimeric product **I-G** are presented in kJ/mol.

Another possible reaction pathway for homodimer **I-G** formation could be observed from intermediates **I-D** and **I-E** (Scheme 8) with the free energy change of −101 kJ/mol for ZnCl_2_ as a metal salt, and −111 kJ/mol if Zn(OAc)_2_ is used. The downside of this reaction path is related to the formation of intermediate **I-D,** which is actually an energetically unfavorable process. For the formation of the latter compound, from intermediate **I-C**, the free energy needed varies between +61 and +81 kJ/mol, depending on the reaction conditions—the metal salt. The following formation of **I-E** is a strongly exergonic process −136 kJ/mol (ZnCl_2_), −132 kJ/mol (Zn(OAc)_2_), and −248 kJ/mol (Cu(OAc)_2_). Even though the formation of intermediate **I-D** is an endergonic process, its formation is not to be excluded since the experimental conditions being used include a temperature of 40 °C and ultrasound irradiation.

### 2.4. Radical Mechanisms

We have also investigated the possibility of the formation of radicals during the reaction process. We have calculated intermediate **I-B** as a triplet biradical structure with Zn and as doublet radical with Cu, coordinated towards the oxygen atoms from the 3-acetylcoumarin **4**. The formation of the Zn-coordinated **I-B** intermediate is an endergonic process: +54 kJ/mol, Scheme 5. However, the formation of the Cu-containing intermediate **I-B** is exergonic: −53 kJ/mol. Even though the intermediate that might be formed with copper is much more energetically favorable, both metals could form the intermediate structure **I-B** (in the case of **I-B-Zn** forming a triplet biradical structure and a doublet structure in the case of **I-B-Cu**), due to the experimental reaction conditions.

The formation of the desired product—structure **I-G**, via the radical mechanism, could occur spontaneously from two molecules of the intermediate **I-B-Zn**, as a homodimerization process, together with one molecule of the metal salt, Scheme 8. The formation of the C-C bond between the monomers occurs spontaneously (∆G = −214 kJ/mol for ZnCl_2_, −163 kJ/mol in the case of Zn(OAc)_2_), which might prove the possibility of the radical mechanism occurring.

Even though the formation of intermediate **I-B** with Cu-atom (**I-B-Cu**, Scheme 5), is much more favorable than the formation of **I-B-Zn**, the desired product **I-G** with Cu-OAc fragments is not stable—the C-C bond dissociates during the geometry optimization process and two monomers are formed again. The cleavage of the C-C bond in the case of Cu as a metal corresponds to the experimental data, where no product is observed during the reaction, even after a long period of time.

From both the experimental data and the quantum-chemical calculations that have been performed, could be concluded that the most plausible reaction pathway includes the formation of biradicals which later form the desired product. The most favorable reaction conditions include the use of THF as a solvent and Zn/Zn(OAc)_2_ as a metal/metal salt pair. In this way, the desired product is obtained with high yields and the reaction time is shorter. The positive effect of the solvent, used in the reaction conditions, could be further confirmed with the computational results which indicate that the dissociation of the metal salts is assisted by the solvent molecules. It can be seen that the most promising results and lowest bond dissociation energies—Zn-ligand, are observed in the case of Zn(OAc)_2_. Moreover, the formation of the homodimer of the 3-acetylcoumarin from the biradical **I-B-Zn** is a spontaneous exergonic process. Even though the formation of the intermediate **I-B-Zn** itself is endergonic, the reaction conditions—ultrasonic irradiation and a temperature of 40 °C could facilitate its formation.

As it is known from experimental data, the homodimer is not observed in the cases which include only Cu as a metal and Cu-salts as reaction conditions. To clarify this result, some of the performed quantum-chemical calculations included ionic intermediates using Cu metal and metal salts, instead of Zn (Scheme 6). In those cases, the formation of the intermediates is an exergonic process, and their formation is favorable and spontaneous, but the formation of the desired product cannot occur (as during the geometry optimization, the C-C bond between the two coumarin monomers cleavages). The obtained results from the quantum-chemical calculations correlate with the experimental data which show the reaction does not occur in those conditions. One of the possible reasons for this is the high stability of the formed intermediates between the 3-acetylcoumarin and the copper.

## 3. Materials and Methods

Ultrasonic irradiation was performed in an Ultrasonic cleaning unit Elmasonic P with a frequency of 37 and 80 kHz and heating (Elma Schmidbauer GmbH). Melting points were determined with a Kofler hot-stage apparatus (Reichert Technologies, New York, NY, USA) and are used without correction. The IR spectra were recorded with a Specord IR 71, IR 75 spectrophotometer (Carl Zeiss, 73447 Oberkochen, Germany). Reactions were monitored by TLC on silica gel 60 F_254_. Column chromatography was carried out on silica gel (Merck 0.043–0.063 mm) (Merck, Kenilworth, NJ, USA) using as eluent *n*-hexane/EtOAc mixture with increasing polarity. All chemical reagents were purchased from Merck and Sigma Aldrich (Taufkirchen, Germany).

### 3.1. General Procedure for the Preparation of 3,3′-Diacetyl-[4,4′-Bichroman]-2,2′-Dione, 5 Using Metals and Metallic Salts

**Method A**—A mixture of 3-acetylcoumarin **4** (0.188 g, 0.001 mol), Zn (0.366 g, 0.0056 mol), (ClCH_2_CO)_2_O (0.410 g, 0.0024 mol) in Et_2_O/THF (10 mL/7 mL) and a catalytic amount of I_2_ was sonicated until the coumarin **4** was consumed (TLC-monitoring), see Table 3. The reaction mixture was poured onto a 2N solution of hydrochloric acid and ice, extracted with chloroform (3 × 20 mL), and the organic extracts were washed several times with saturated solution of NaHCO_3_ and then dried with anhydrous sodium sulfate. The solvent was evaporated and 3 mL Et_2_O and 1 mL acetone were added to the residue. The resulting mixture was left in a fridge overnight. Compound **5** was obtained as a solid. After the filtration of the crystals, the solvent of the mother liquor was evaporated and the residue was purified by column chromatography using *n*-hexane/EtOAc as an eluent system.

**Method B.1**—A mixture of 3-acetylcoumarin **4** (0.188 g, 0.001 mol), Zn (0.366 g, 0.0056 mol), ZnCl_2_ (0.204 g, 0.0015 mol) and a catalytic amount of I_2_ was sonicated until the coumarin 4 was consumed (TLC-monitoring), see Table 3. The reaction mixture was worked up as in **Method A**, except that the reaction mixture was poured onto 10 mL concentrated hydrochloric acid.

**Method B.2**—Performed as **Method B.1**; however, the used metallic salt ZnCl_2_ was 0.408 g (0.003mol).

**Method B.3**—Using procedure for **Method B.1**; however, the used metallic salt was Zn(CH_3_COO)_2_ × 2H_2_O (0.329 g, 0.0015 mol).

**Method B.4**—In a manner similar as **Method B.1**; however, the used Zn(CH_3_COO)_2_ × 2H_2_O was 0.659g (0.003 mol).

**Method B.5**—Performed as **Method B.1**; however, the used metallic salt was Cu(CH_3_COO)_2_ × H_2_O (1.118 g, 0.0056 mol).

**Method B.6**—Using procedure for **Method B.1**; however, the used metallic salt was CuBr_2_ (0.223 g, 0.001 mol).

**Method B.7**—Performed as **Method B.1**; however, the used metallic salt was Co(CH_3_COO)_2_ (0.265 g, 0.0015mol).

**Method B.8**—Using procedure for **Method B.1**; however, the used metallic salt was CoCl_2_ (0.195 g, 0.0015mol).

**Method B.9**—In a manner similar as **Method B.4**; however, K_2_S_2_O_8_ (0.405 g, 0.0015 mol) was added.

**Method B.10**—Using procedure for **Method B.1**; however, ZnO (0.244 g, 0.003 mol) was used.

**Method B.11**—Performed as **Method B.1**; however, the used metal and metallic salt were Cu (0.355 g, 0.0056 mol) and Zn(CH_3_COO)_2_ × 2H_2_O (0.329 g, 0.0015 mol).

**Method B.12**—Performed as **Method B.11**; however, the used metallic salt was Cu(CH_3_COO)_2_ × H_2_O (0.299g, 0.0015 mol).

**Method B.13**—Using procedure for **Method B.1**; however, the metal and the metallic salt were Fe (0.312g, 0.0056 mol) and Zn(CH_3_COO)_2_ × 2H_2_O (0.329 g, 0.0015 mol).

**Method B.14**—The reaction was carried out as in **Method B.1**, but the metal and the metallic salt were Mg (0.134 g, 0.0056 mol) and MgCl_2_ (0.285 g, 0.003 mol).

### 3.2. Procedure for Optimization of the Used Amount of Zinc in the Reaction

**Method C.1**—A mixture of 3-acetylcoumarin **4** (0.188 g, 0.001 mol), Zn (0.065 g, 0.001 mol), Zn(CH_3_COO)_2_ × 2H_2_O (0.659g, 0.003 mol) and a catalytic amount of I_2_ in THF (10 mL) was sonicated until the coumarin **4** was consumed (TLC-monitoring), see Table 5. The reaction mixture was poured onto a 10 mL concentrated hydrochloric acid, extracted with dichloromethane (5 × 10 mL), and the organic extracts were washed with water (2 × 15 mL) and saturated solution of NaCl (2 × 15 mL). The extract was dried with anhydrous sodium sulfate and the solvent was evaporated. Et_2_O (3 mL) and acetone (1 mL) were added to the residue and the resulting mixture was left in a fridge overnight. Compound **5** was obtained as a solid. After the filtration of the crystals, the solvent of the mother liquor was evaporated, and the residue was purified by column chromatography using *n*-hexane/EtOAc as an eluent system.

**Method C.2**—Using procedure for **Method C.1**; however, the quantity of the metal was 0.131 g Zn (0.002 mol).

**Method C.3**—Performed as **Method C.1**; however, the used metal was Zn (0.196 g, 0.003 mol).

**Method C.4**—Using procedure for **Method C.1**; however, the quantity of the metal was Zn (0.262 g, 0.004 mol).

**Method C.5**—Using procedure for **Method C.1**; however, the quantity of the metal was 0.282 g Zn (0.005 mol).

**Method C.6**—Performed as **Method C.1**; however, the used metal was Zn (0.366 g, 0.0056 mol).

**Method C.7**—Performed as **Method C.1**; however, the used metal was Zn (0.392 g, 0.006 mol).

### 3.3. Procedure for Optimization of the Used Amount of Zn(CH_3_COO)_2_ × 2H_2_O for the Reaction

**Method D.1**—A mixture of **4** (0.188 g, 0.001 mol), Zn (0.262 g, 0.004 mol), Zn(CH_3_COO)_2_ × 2H_2_O (0.219 g, 0.001 mol) and a catalytic amount of I_2_ in THF (10 mL) was sonicated until the consumption of the coumarin **4** (TLC-monitoring), see Table 6. The reaction mixture was poured onto a 10 mL concentrated hydrochloric acid, extracted with dichloromethane (5 × 15 mL), and the organic extracts were washed with water (2 × 15 mL) and saturated solution of NaCl (2 × 15 mL). The extract was dried with anhydrous sodium sulfate and the solvent was evaporated. Et_2_O (3 mL) and acetone (1 mL) were added to the residue and the resulting mixture was left in a fridge overnight. Compound **5** was obtained as a solid. After the filtration of the crystals, the solvent of the mother liquor was evaporated and the residue was purified by column chromatography using *n*-hexane/EtOAc as an eluent system.

**Method D.2**—Performed as **Method D.1**; however, the used Zn(CH_3_COO)_2_ × 2H_2_O was 0.439 g (0.002 mol).

**Method D.3**—Performed as **Method D.1**; however, the used Zn(CH_3_COO)_2_ × 2H_2_O was 0.658 g (0.003 mol).

**Method D.4**—Performed as **Method D.1**; however, the used Zn(CH_3_COO)_2_ × 2H_2_O was 0.878 g (0.004 mol).

**3,3′-Diacetyl-[4,4′-bichroman]-2,2′-dione, 5**—The product was isolated as white crystals, mp = 196–198 °C. **IR** (nujol): ν = 1595, 1645, 1450 cm^−1^ [17].

### 3.4. Computational Details

The quantum-chemical modeling was performed with the density functional theory (DFT) [33,34,35,36] using the Gaussian09 suite of programs [37]. For the calculations the hybrid B3LYP exchange-correlation functional was used [38,39,40,41], coupled with 6-31++G** basis set and polarizable continuum (PCM) [42] model. The PCM model describes the solvent as a homogeneous dielectric medium that is polarized around the solvated molecule. The chosen solvent for the calculations is THF in order to have better correlation with the experimental results. In order to check the accuracy of the chosen functional and basis set, we have performed benchmarking, using post-HF MP2 [43,44,45,46,47] method and correlation consistent triple-zeta basis set cc-pTZV. To further prove the accuracy of the method that was used, we have calculated some of the reaction steps with D3 dispersion correction [48]. Both methodologies do not change the found trends in the study qualitatively. We have also used relaxed potential surface energy scan, along certain reaction coordinates to investigate the bond dissociation energy in some of the reaction steps. To acquire the thermodynamic parameters of the investigated systems and also to ensure that they are in true minima, we have calculated the IR frequencies for all structures.

## 4. Conclusions

By means of the DFT modeling and experimental results the homodimerization process of the 3-acetylcoumarin **4** was investigated. Two reaction pathways were considered: ionic and radical. Under ultrasound irradiation with combination of Zn and zinc salt an electron transfer process occurs resulting in activation of the coumarin specie. The reaction is sensitive to several factors as the nature of the reactant, the amount of the used metal and metal salts which played a major role for the outcome of the homodimerization reaction. The formation of radical intermediates with Zn and Cu metals were evaluated using their enthalpies. Thus, the main experimental observations clarified the necessity of both Zn and Zn-salt in the reaction mixture and answered the question why the reaction occurs in the presence of Zn/Zn-salt and not with Cu/Cu-salt. The presence of Zn metal is crucial for coumarin biradical generation which is intermediate for the biscoumarin formation. Such biradical complex cannot be formed with Cu as metal. The zinc salt is important to facilitate the dimerization process. Furthermore, the frequency and the amplitude of the ultrasonic bath was identified as significant variables for the activation of the zinc metal and reaction initiation.

The dimeric product of 3-acetylcoumarin was obtained in quantitative yields. The different solubility of zinc (II) salts, compared to chloroacetic anhydride, used in our previous study, required an optimization of the organic solvents where tetrahydrofuran was found as the most suitable solvent for the studied electron-transfer reactions. The theoretical data followed the experimental findings and clarified that the reaction could not be initiated in diethyl ether. On the base of bond dissociation energy between the metal and metal salts the assistance of the solvent molecules was confirmed. The most promising results and lowest Zn-ligand bond dissociation energies were observed in the case of Zn(OAc)_2_. These findings are in line with the experimental observations, as the reaction takes place for shorter time and higher yield in Zn(OAc)_2_ × 2H_2_O than in ZnCl_2_.

The experimental data and the quantum-chemical calculations demonstrated that the most plausible reaction pathway includes the formation of biradicals which later form the desired product. The most favorable reaction conditions include the use of THF as a solvent and Zn/Zn(OAc)_2_ as a metal/metal salt pair. The positive effect of the solvent facilitated the formation **I-B-Zn** to the final product **5.**

## Figures and Tables

**Table 1:** Reaction of **4** and chloroacetic anhydride by varying the ratio between the reactants.

Reaction Conditions	Reaction Time	Yield
Ratio Coumarin 4:(ClCH_2_CO)_2_O		
1:0.5	45 min	66%
1:1	35 min	77%
1:1.5	10–15 min	90%
1:2.4 *	10 min	92%
1:3	5 min	82%

* Previous results [17], Zn:(ClCH_2_CO)_2_O:coumarin 5.6:2.4:1, THF/Et_2_O (**Method A**), 20 kHz for 10 min, 92% yield.

**Table 2:** Reaction of coumarin **4** with zinc and (ClCH_2_CO)_2_O in different frequencies and amplitudes.

37 kHz, Sweep Mode			80 kHz, Sweep Mode		
Amplitude	Reaction Time	Yield	Amplitude	Reaction Time	Yield
100%	20 min	74%	100%	60 min	71%
60%	10 min	88%	60%	150 min	85%
30%	5–10 min	90%	30%	90 min	70%

**Table 3:** Evaluating the combination metal and metal salts as new conditions for the homodimerization reaction.

Method	Metal	MX		Reaction Time	Yield	Ratio *meso-* to *d/l*
		Initiator	Amount			
A *	Zn	(ClCH_2_CO)_2_O	2.4 eqv.	10 min	92%	1:0.65
**B.1**	Zn	ZnCl_2_	1.5 eqv.	60 min	57%	1:0.58
**B.2**	Zn	ZnCl_2_	3.0 eqv.	30 min	63%	1:0.61
**B.3**	Zn	Zn(OAc)_2_ × 2H_2_O	1.5 eqv.	60 min	89%	1:0.54
**B.4**	Zn	Zn(OAc)_2_ × 2H_2_O	3.0 eqv.	30 min	97%	1:0.55
**B.5**	Zn	Cu(OAc)_2_ × H_2_O	5.6 eqv.	60 min	-	
**B.6**	Zn	CuBr_2_	1.0 eqv.	4 days	-	
**B.7**	Zn	Co(OAc)_2_	1.5 eqv.	60 min	-	
**B.8**	Zn	CoCl_2_	1.5 eqv.	1 day	60%	1:0.53
**B.9**	Zn	Zn(OAc)_2_ × 2H_2_O/K_2_S_2_O_8_	3.0 eqv./1.5 eqv.	390 min	86%	
**B.10**	Zn	ZnO	3.0 eqv.	120 min	-	
**B.11**	Cu	Zn(OAc)_2_ × 2H_2_O	1.5 eqv.	240 min	-	
**B.12**	Cu	Cu(OAc)_2_ × H_2_O	1.5 eqv.	120 min	-	
**B.13**	Fe	Zn(OAc)_2_ × 2H_2_O	1.5 eqv.	360 min	-	
**B.14**	Mg	MgCl_2_	3.0 eqv.	120 min	-	

* Previous results [17], Zn:(ClCH_2_CO)_2_O:coumarin 5.6:2.4:1, THF/Et_2_O (**Method A**).

**Table 4:** The influence of the reaction media on the outcome of the dimerization process.

Solvent	Reaction Time	Yield
THF:Et_2_O (3.5:5)	30 min	97%
C_6_H_6_	120 min	N/A
CH_2_Cl_2_	80 min	Complexed mixture
THF	10 min	62%
Et_2_O	135 min	N/A

**Table 5:** Optimization of the applied amount of metallic Zn.

Method C	Reaction Conditions	Reaction Time	Yield
	Ratio Coumarin 4:Zn *		
**C.1**	1:1	90 min	65%
**C.2**	1:2	90 min	64%
**C.3**	1:3	10–15 min	71%
**C.4**	1:4	10 min	77%
**C.5**	1:5	10 min	69%
**C.6**	1:5.6	10 min	62%
**C.7**	1:6	10 min	67%

* The reactions were carried out in the presence of 3 eqv. Zn(OAc)_2_ × 2H_2_O in THF.

**Table 6:** Optimization of the applied amount of Zn(OAc)_2_ × 2H_2_O in the reaction.

Method D	Reaction Conditions	Reaction Time	Yield
	Ratio Coumarin 4:Zn(OAc)_2_ × 2H_2_O *		
**D.1**	1:1	65 min	-
**D.2**	1:2	30–40 min	66%
**D.3**	1:3	10–15 min	77%
**D.4**	1:4	10 min	72%

* The reactions were carried out in the presence of 4 eqv Zn in THF.

## Data Availability

Not applicable.

## Supplementary Materials

The following supporting information can be downloaded at: https://www.mdpi.com/article/10.3390/molecules27217228/s1, Table S1: Relative stability of the possible complexes that could be formed between the 3-acetylcoumarin and ZnCl_2_ molecule; Table S2: Benchmarking calculations for selected reaction steps in vacuum. All energies are in (kJ/mol); Table S3: Benchmarking calculations for selected reaction steps in PCM(THF). All energies are in (kJ/mol); Figure S1: Formation of intermediate **I-E** from intermediate **I-C**, in the presence of Zn and ZnCl_2_, via Grotos-like mechanism; Figure S2: Full reaction scheme of transformation of 3-acetylcoumarin to a dimer structure; Figure S3: Dissociation of acetate anion from Zn(OAc)_2_.2H_2_O. (a) Initial geometry of the structure; (b) Structure in the first minimum; (c) Structure in second maximum (distance between Zn atom and C-atom from acetyl group—3.71 Å); (d) Structure in minimum—formation of the 6-membered cycle; Figure S4: Dissociation of acetate anion from Zn(OAc)_2_.2H_2_O. (a) Initial geometry of the structure; (b) Structure in the first minimum; (c) Structure in second maximum (distance between Zn atom and C-atom from acetyl group—3.71 Å); (d) Structure in minimum—formation of the 6-membered cycle; Table S4: Calculations for selected reaction steps in PCM(THF), using D3 dispersion correction with B3LYP/6-31++G**, compared to the calculations without the D3 correction. All energies are in (kJ/mol).

## Sample Availability

Samples of the compounds are available from the authors.
