# Supplementary material for: Experimental and Theoretical Study on the Homodimerization Mechanism of 3-Acetylcoumarin
Source: Molecules. 2022 Oct 25;27(21):7228. doi: 10.3390/molecules27217228 (PMC9655359; doi:10.3390/molecules27217228)
Supplement: Supplementary file 1 [file molecules-27-07228-s001.zip › molecules-1974875-supplementary.pdf]

Supplementary Materials

Experimental and Theoretical Study on the Homodimerization Mechanism of 3-Acetylcoumarin

Kristina B. Simeonova, Ana I. Koleva, Anna-Mariya R. Zlatanova, Nevena I. Petkova-Yankova*, Hristiyan A. Aleksandrov, Petko St. Petkov and Rositca D. Nikolova

Faculty of Chemistry and Pharmacy, Sofia University "St. Kliment Ohridski", 1 James Bourchier Blvd, 1164 Sofia, Bulgaria
* Correspondence: nipetkova@chem.uni-sofia.bg; Tel.: +359-2-81-61-249

Table S1. Relative stability of the possible complexes that could be formed between the 3-acetylcoumarin and $ZnCl_2$ molecule.

**Structure I-C**					
**A**	**B**	**C**	**D**	**E**	**F**
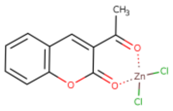	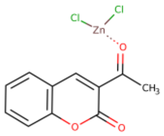	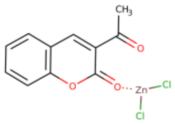	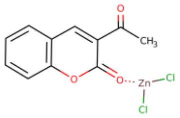	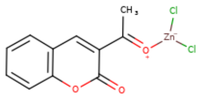	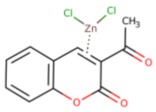
0 kJ/mol	+23 kJ/mol	+26 kJ/mol	+15 kJ/mol	+28 kJ/mol	$ZnCl_2$ desorbs from the double C=C bond

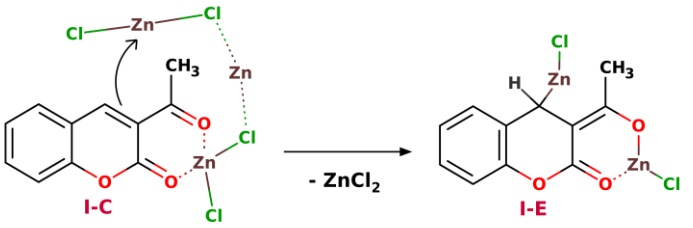

Figure S1. Formation of intermediate **I-E** from intermediate **I-C**, in the presence of Zn and $ZnCl_2$, via Grotos-like mechanism.

In order to check whether the B3LYP functional and 6-31++G** basis set are accurately describing the investigated models, we have done a benchmarking, using higher level of theory and basis sets, for some of the intermediates that are potentially being formed during the ionic mechanism. The calculations were conducted with and without the PCM model, that has been used throughout the calculations. When a different basis set is used – cc-pTZV instead of 6-31++G**, the relative electronic energies and the tendencies stay qualitatively the same. (**Table S2**) The formation of intermediate **I-C** is -83 kJ/mol and -84 kJ/mol for 6-31++G** and cc-pTZV respectively, the formation of **I-D** from intermediate **I-C** is an endothermic process in both cases +69 kJ/mol (6-31++G**) and +75 kJ/mol (cc-pTZV) and the formation of **I-E** from both **I-C** and **I-D** is still an exothermic process. In this case, when only the basis set is changed (to cc-pTZV), the relative energy is slightly higher, compared to the chosen basis set – 6-31++G**, but the tendencies don't change.

When the same relative energies are compared to MP2/cc-pTZV method, an increase in the absolute values in almost all cases is observed. Nevertheless, the tendencies remain the same – all of the calculated intermediates are to be formed spontaneously, except the formation of intermediate **I-D**, which is, again, an endothermic process.

Table S2. Benchmarking calculations for selected reaction steps in vacuum. All energies are in [kJ/mol].

Formation of intermediates*	B3LYP/6-31++G**	B3LYP/cc-pTZV	MP2/cc-pTZV
**4 → I-C**	-83	-84	-105
**I-C → I-D**	+69	+75	+109
**I-C → I-E**	-33	-34	-9
**I-D → I-E**	-103	-109	-118

* See **Scheme 5, 6, 7** in main text.

Table S3. Benchmarking calculations for selected reaction steps in PCM(THF). All energies are in [kJ/mol].

Formation of intermediates*	B3LYP/6-31++G**	MP2/cc-pTZV
**4 → I-C**	-27	-45
**I-C → I-D**	+78	+120
**I-C → I-E**	-171	-191
**I-D → I-E**	-93	-20

* See **Scheme 5, 6, 7** in main text.

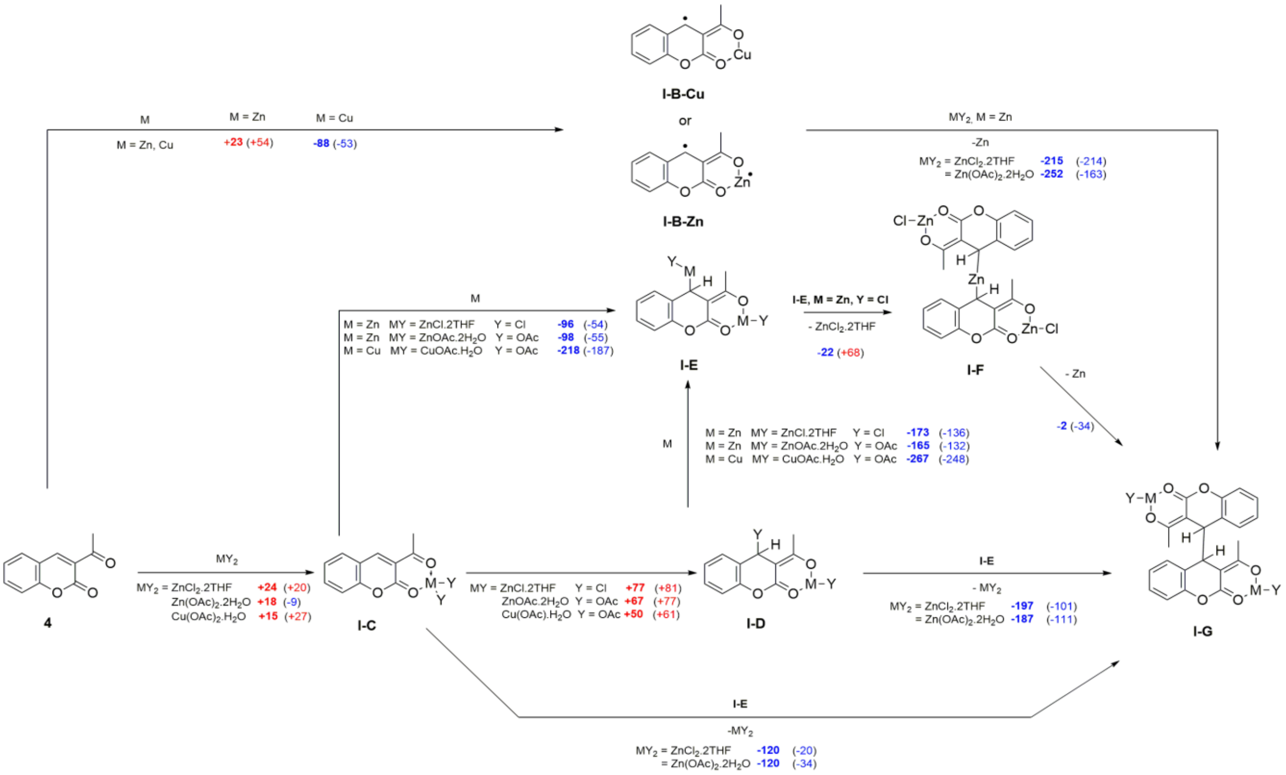

Figure S2. Full reaction scheme of transformation of 3-acetylcoumarin to a dimer structure.

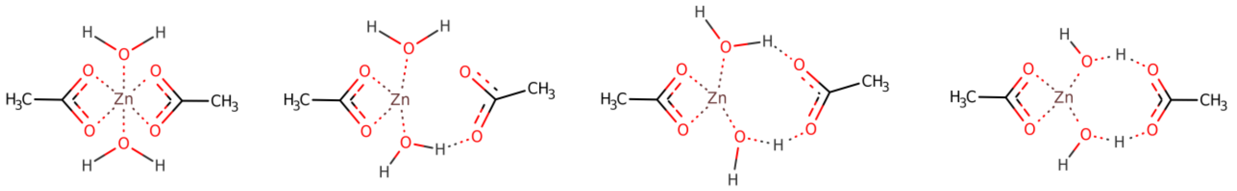

(a) (b) (c) (d)

Figure S3. Dissociation of acetate anion from Zn(OAc)2.2H2O. **(a)** Initial geometry of the structure; **(b)** Structure in the first minimum; **(c)** Structure in second maximum (distance between Zn atom and C-atom from acetyl group – 3.71 Å); **(d)** Structure in minimum – formation of the 6-membered cycle.

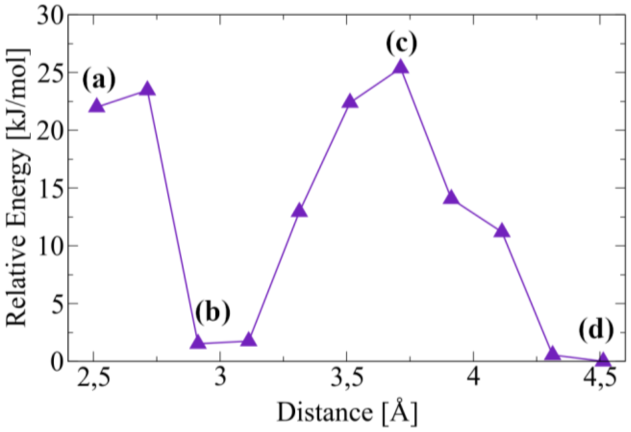

Figure S4. Dissociation of acetate anion from Zn(OAc)2.2H2O. **(a)** Initial geometry of the structure; **(b)** Structure in the first minimum; **(c)** Structure in second maximum (distance between Zn atom and C-atom from acetyl group – 3.71 Å); **(d)** Structure in minimum – formation of the 6-membered cycle.

Table S4. Calculations for selected reaction steps in PCM(THF), using D3 dispersion correction with B3LYP/6-31++G**, compared to the calculations without the D3 correction. All energies are in [kJ/mol].

Formation of intermediates*	Enthalpies			Gibbs free energies		
	Without D3	With D3	D3 contribution**	Without D3	With D3	D3 contribution**
**4 → I-B**	+23	+16	-7	+54	+48	-6
**4 → I-C**	+24	+55	31	+20	+12	-8
**I-C → I-D**	+77	+71	-6	+81	+77	-4
**I-C → I-E**	-96	-108	-12	-54	-66	-12
**I-D → I-E**	-173	-179	-6	-136	-143	-7
**I-B → I-G**	-215	-245	-30	-214	-242	-28
**I-C → I-G**	-120	-216	-96	-20	-106	-86

* See **Scheme 5, 6, 7** in main text.

** E(with D3 correction)-E(without D3 correction)
